# Patients’ perceptions on the impact of coffee consumption in inflammatory bowel disease: friend or foe? – a patient survey

**DOI:** 10.1186/s12937-015-0070-8

**Published:** 2015-08-12

**Authors:** Christiane Barthel, Sandra Wiegand, Sylvie Scharl, Michael Scharl, Pascal Frei, Stephan R. Vavricka, Michael Fried, Michael Christian Sulz, Nico Wiegand, Gerhard Rogler, Luc Biedermann

**Affiliations:** 1 Division of Gastroenterology and Hepatology, University Hospital Zurich, Raemistrasse 100, 8091 Zurich, Switzerland; 2 Division of Gastroenterology and Hepatology, Robert-Bosch-Hospital Stuttgart, Stuttgart, Germany; 3 Division of Gastroenterology & Hepatology, Klinik St. Anna, Luzern, Switzerland; 4 Zurich Center for Integrative Human Physiology, University of Zurich, Zurich, Switzerland; 5 Division of Gastroenterology & Hepatology, Clinic Bethanien, Zurich, Switzerland; 6 Division of Gastroenterology & Hepatology, Triemli Hospital, Zurich, Switzerland; 7 Division of Gastroenterology & Hepatology, Kanton Hospital St. Gallen, St. Gallen, Switzerland

## Abstract

**Background:**

Environmental factors are an integral component in the pathogenesis of inflammatory bowel disease (IBD). There is an increasing interest in nutritive components. While the potential disease-modifying role of coffee has been intensively investigated in a variety of gastrointestinal diseases, the data on the potential impact on IBD is very limited. We aimed to determine the patients’ perspective on coffee consumption in IBD.

**Methods:**

We conducted a questionnaire among IBD patients in Switzerland, assessing key questions regarding coffee consumption. Descriptive statistics including chi square testing were used for analysis of questionnaire data.

**Results:**

Among a total of 442 patients 73 % regularly consume coffee. 96 % of patients attributing a positive and 91 % of patients attributing no impact of coffee intake on IBD regularly drink coffee and surprisingly even 49 % of those patients that assign a negative impact on disease symptoms. Among those patients refraining from regular coffee intake 62 % are convinced that coffee adversely influences intestinal symptoms, significantly more in Crohn’s disease (CD) than in ulcerative colitis (UC) (76 % vs. 44 %, p = 0.002). In total, 38 % of all study subjects suppose that coffee has an effect on their symptoms of disease, significantly more in CD (54 %) compared to UC patients (22 %, p < 0.001). Moreover, while 45 % of CD patients feel that coffee has a detrimental influence, only 20 % of UC patients share this impression (p < 0.001).

**Conclusion:**

Two thirds of IBD patients regularly consume coffee. More than twice as many CD compared to UC patients attribute a symptom-modifying effect of coffee consumption, the majority a detrimental one. However, this negative perception does not result in abstinence from coffee consumption.

**Electronic supplementary material:**

The online version of this article (doi:10.1186/s12937-015-0070-8) contains supplementary material, which is available to authorized users.

## Background

A variety of environmental and genetic factors, an altered intestinal microbiota, and aberrant immune responses have been considered as the major etiologic components in inflammatory bowel disease (IBD), a group of chronic inflammatory diseases of the gastrointestinal tract (GIT), including Crohn’s disease (CD) and ulcerative colitis (UC) [1, 2].

The effect of smoking – the most profoundly investigated environmental factor in IBD - has already been known for decades [3]. However, many others have been explored, such as oral contraceptives [4, 5], appendectomy [6, 7], breastfeeding [8], infections [9–11], antibiotics [12, 13] and non-steroidal anti-inflammatory drugs (NSAIDs) [14], and diet [15].

Only in recent years the potential relationship between nutritional intake and IBD has been increasingly explored [15, 16]. A recent large prospective study revealed a protective effect of high dietary fiber intake, above all from fruit, for CD [17]. While the development of CD was associated with the intake of total fats and consumption with sugar and/or sweeteners [18, 19], a similar association was found between UC and monounsaturated and polyunsaturated fat consumption [20].

Coffee as one of the most popular beverages worldwide and especially in the Western countries has recently gained attention, when its positive effects were described for a variety of chronic diseases, such as liver diseases (including hepatitis C and non-alcoholic fatty liver disease) [21–24], type 2 diabetes mellitus [25, 26], Parkinson’s disease [27], constipation [28], and cancer [29, 30]. The probably largest prospective study on this topic showed inverse associations of coffee consumption with overall mortality and specifically with death due to heart disease, respiratory disease, stroke, injuries and accidents, diabetes, and infections [31].

Coffee represents a highly complex mixture of different compounds, containing more than a thousand different chemical constituents, including carbohydrates, lipids, nitrogenous compounds, vitamins, minerals, alkaloids and phenolic compounds and providing significant amounts of chlorogenic acid and caffeine [32]. However, in view of their concentration in the brew, previous studies on detection of causal compounds or metabolites in the body and physiological effects, only three highly abundant ingredients appear to be of importance: caffeine (1,3,7-trimethylxanthine, a purine alkaloid) [32], the diterpene alcohols (cafestol and kahweol) as well as chlorogenic acid and other polyphenols [33, 34] Regarding the GIT, the effects of coffee and caffeine are increasingly explored. At typical daily intake levels, caffeine appears to exert biological effects through the antagonism of the A1 and A2A subtypes of the adenosine receptor [35]. Adenosine is an endogenous neuromodulator with mostly inhibitory effects, and adenosine antagonism by caffeine that results in effects that are generally stimulatory [36]. Moreover, coffee has prebiotic effects but also antibacterial activity inducing alterations in intestinal microbiota such as a decrease in E. coli and Clostridium spp. as well as an increase in Lactobacillus spp.and Bifidobacterium spp. [37, 38]. In addition, effects on gastrointestinal motility including stimulation of colonic motor activity [39] and increase in rectal tone [40] have been described. A recent patient survey indicated a protective effect of coffee consumption on the risk of developing primary sclerosing cholangitis [41].

Despite the plurality of the aforementioned findings on coffee in gastrointestinal medicine and despite the fact that coffee is one of the most ubiquitously consumed beverages all over the world, the potential role of coffee intake on the course of disease in IBD has not been investigated so far. Using a patient survey in a large collective of IBD patients in Switzerland, we aimed to investigate the consumption behavior and perception among IBD patients towards coffee.

## Methods

### Study cohort and questionnaire

This questionnaire-based patient survey was conducted among patients with known CD and UC or IBD unclassified (IBDU) from the Swiss Crohn’s and Colitis Patients Association (SMCCV,) with roughly 2000 members. Between December 2012 and May 2013*,* all members were informed about the questionnaire in the regularly published print journal of the SMCCV as well as on the SMCCV homepage and were invited to participate via conventional mail or web-based on a voluntary basis without receiving any financial recompensation. Inclusion in the survey ended in May.2013. The study participants were asked to answer a short questionnaire consisting of 4 questions on their consumption and perception of coffee regarding possible impact on their symptoms (*the full questionnaire is provided in the appendix,* Additional file 1: Table S1). Patients’ data were anonymized.

### Statistics

The answers were analyzed by means of descriptive statistics. Comparison between CD and UC patients were performed using chi square testing, p-values of less than 0.05 were considered statistically significant. The statistical analyses were performed using SPSS (Version 21; IBM, Armonk, NY, USA).

## Results

A total of 497 IBD patients representing nearly 25 % of all members of the SMCCV answered our survey, including 330 (66.4 %) patients with CD, 157 (31.6 %) with UC, and 10 (2 %) with IBD unclassified (IBDU). Due to inappropriate responses, 55 questionnaires had to be excluded from further analysis.

### Coffee drinkers among IBD patients

**Fig. 1:**
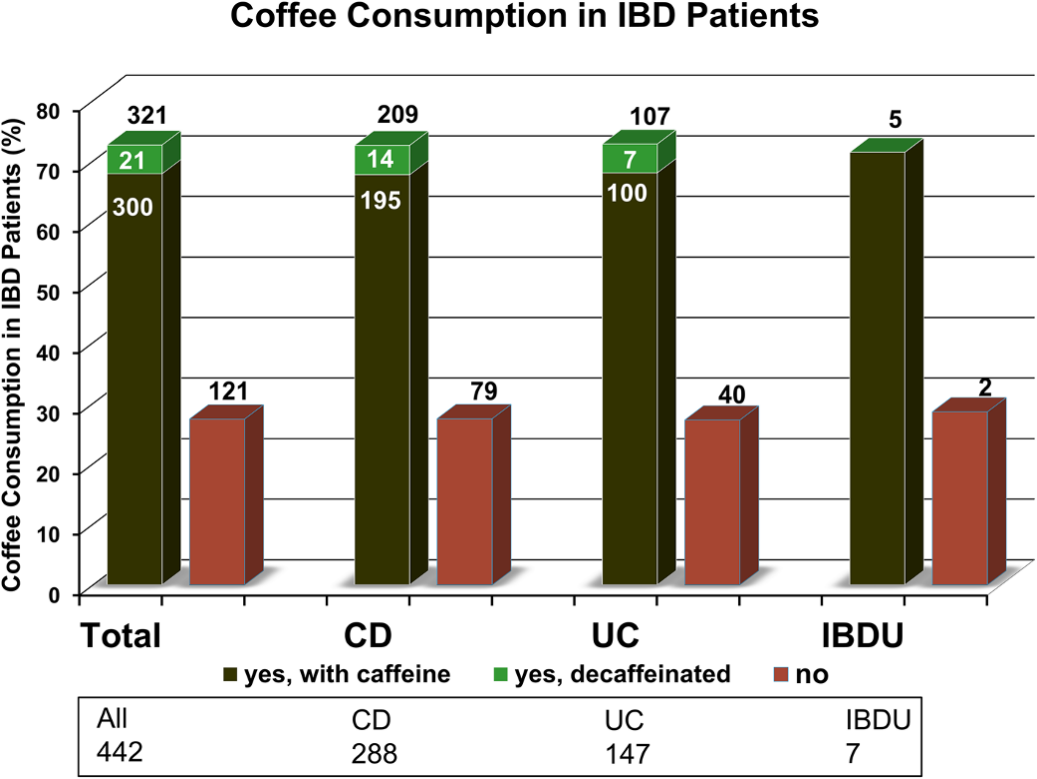
Coffee consumption in 442 IBD patients. Almost three quarters of patients (72.6 %) regularly consume coffee (93.5 % caffeinated coffee, 6.5 % decaffeinated coffee). This distribution appears very similar in CD, UC and IBDU (209 of 288, 72.6 % of CD, 107 of 147, 72.8 % of UC and 5 of 7, 71.4 % of IBDU patients; IBD: Inflammatory bowel disease, CD: Crohn’s disease, UC: ulcerative colitis, IBDU: IBD unclassified)

Out of 442 patients included for data analysis, 321 (72.6 %) patients regularly consumed coffee (Fig. 1). This distribution appeared very similar in the subtypes of IBD (CD 72.6 %, UC 72.8 %, IBDU 71.4 %; Fig. 1).

The vast majority of regular coffee drinkers (300/321; 93.5 %) preferred coffee with caffeine, while only 6.5 % (21/321) of patients consumed coffee without caffeine. Among IBD subtypes there were no significant differences between patients who drank coffee with or without caffeine, as well as between those who negated drinking coffee regularly: 67.9 % of CD patients (195/288), 67.7 % (195/288) of patients with UC, and 71.4 % (5/7) of patients with IBD unclassified declared drinking regularly caffeinated coffee, while roughly 5 % in each patient group preferred decaffeinated coffee, and about 28 % did not consume coffee at all or only unregularly (Fig. 1).

### Reasons not to consume coffee regularly

**Fig. 2:**
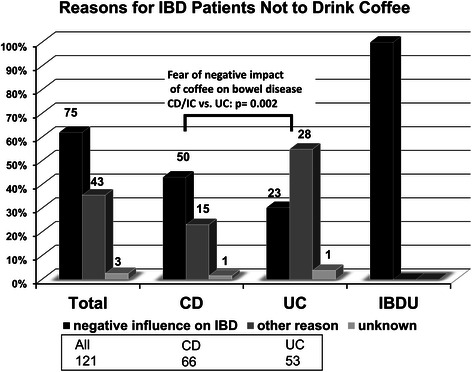
Reasons for 121 IBD patients not to drink coffee. 60 % of those patients mentioned that they fear negative impact on their bowel disease. 30 % mentioned “other reasons” meaning any reason apart from the former reason. The analysis for the separate IBD subgroups show, that,compared to UC patients, both CD and IBDU patients much more often stated not drinking coffee because they experience a negative influence of coffee on their symptoms of disease

Those patients refraining from regular coffee intake were asked for their reason(s) to disclaim coffee. Overall, nearly two thirds (75/121) 62 %) of IBD patients were convinced that consuming coffee would have a negative impact on their intestinal symptoms, while 35.5 % (43/121) referred to “any other reasons” for not drinking coffee, and 2.5 % (3/121) of the patients stated that they did not know why they passed on coffee (Fig. 2). Of note, significant differences between the IBD subtypes were seen. While the majority of patients with CD and IBDU (together 76.4 % 52/68) found that coffee had a negative effect on their disease, only 44.4 % of UC patients (23 of 53) shared this view (p = 0.002).

### IBD patients’ perception of coffee on their bowel disease

**Fig. 3:**
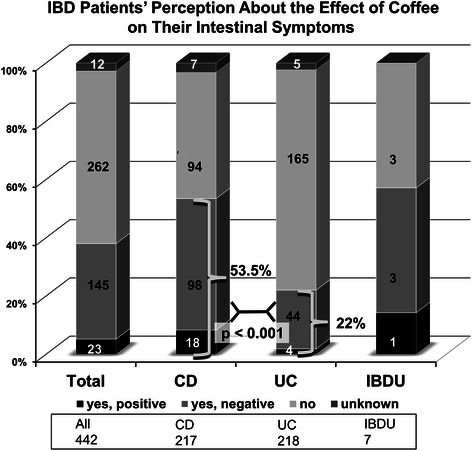
IBD patients’ perception about the effect of regular coffee consumption on their intestinal symptoms. Significantly more patients with CD think that coffee has an overall impact on their bowel disease, compared to UC patients as depicted by brackets combining patients attributing a positive and a negative effect. Among UC patients, the majority (about three quarters of patients) do not think that coffee has any influence at all on their symptoms. Uniformly among all IBD subtypes, if an impact of coffee on disease symptoms is attributed by patients, only a small minority of patients experience a positive influence

All participants (i.e. coffee-drinkers and non-coffee-drinkers) were asked to give their general opinion whether regular coffee intake exerts a positive, negative or no influence at all on their bowel symptoms. In total 38 % of IBD patients (168/442) assumed that coffee does have an overall effect on their symptoms, significantly more in CD than UC (53.5 % vs 22 %; p < 0.001, Fig. 3). Strikingly and highly significant, more than twice as many CD patients (45.2 %) felt that coffee negatively influences their course of disease by worsening intestinal symptoms, as compared to only 20.2 % of UC patients (p < 0.001). Among CD roughly an equal amount of patients (43.3 %) as those stating an adverse influence of coffee did not attribute any effect on coffee on the course of their condition at all. This fraction of patients not identifying any disease modifying effect of coffee intake on the course of their IBD is significantly higher in UC (75.7 %; p < 0.001).

**Fig. 4:**
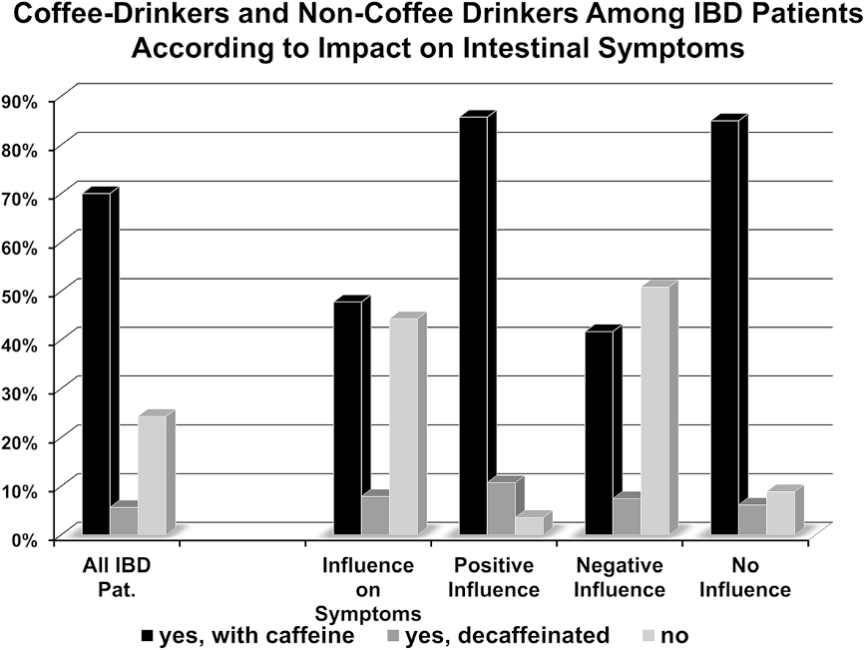
Regular coffee consumption according to the attributed impact on IBD symptoms. While it is not surprising, that the fraction of patients regularly drinking coffee is high in those patients identifying a positive impact and also no impact of coffee on their IBD, the fact that almost every second patient in the group identifying a negative influence of coffee nevertheless regularly consumes this beverage is noteworthy

We next analyzed whether the individual experience on the effect of coffee on the disease course had an impact on coffee consumption. Therefore, we stratified current coffee consumption according to patients’ self-reported impression on the impact of coffee intake on the course of their disease. As expected, significantly more patients, attributing a positive impact to coffee report regular coffee intake compared to their counterparts attributing a negative effect (p < 0.001). However, there is a high fraction of patients that regularly consumed coffee (49.1 % in total; 41.7 % and 7.4 % with and without caffeine, respectively) even in the subgroup of IBD patients that assigned a negative impact of coffee consumption on their symptoms (Fig. 4).

## Discussion

This questionnaire-based patient survey investigated the consumption behavior and perception among 442 IBD patients of the Swiss Crohn’s and Colitis Patient Association towards coffee. While there has been long-standing extensive research on the role of environmental factors, such as smoking, childhood infectious diseases and antibiotic intake [9–13], only in recent years a growing number of investigations shed some light on the potential role of dietary factors on IBD pathogenesis and their potential disease modifying effect [15, 16]. Interestingly, despite the fact that coffee is one of the most ubiquitous consumed beverages all over the world [31] containing a magnitude of ingredients with the potential to exert a wide spectrum of biological effects, there is only very scant data on the interrelation of coffee consumption and IBD. To the best of our knowledge, this is the first study in humans focusing on coffee consumption and IBD patients’ perception regarding their clinical bowel symptoms.

One of the key findings of our survey is the fact, that more than two thirds of all IBD patients regularly consume coffee. While the general per capita consumption of coffee in Switzerland with 7.85 kg per year is among the highest in Europe [42]*,* there is no authoritative data on percentages of the general population in Switzerland regularly consuming coffee. However, the 72.6 % of Swiss IBD patients consuming coffee appear to be in a comparable dimension with recent data from the general population: 83 % of US adults state to drink coffee, 63 % and 75 % declare to consume it daily and at least once per week, respectively [43].

Interestingly, while the subtype of IBD does not appear to be associated with coffee consumption overall, there are significant differences in the perceived effect of coffee on intestinal symptoms between patients with CD and UC. Firstly, the fraction of patients attributing any disease-modifying capacity of coffee at all is more than twice as high in CD compared to UC (53.5 % vs. 22 %, respectively). Secondly, in both IBD subtypes the vast majority of those patients attributing a disease-modifying effect of coffee identified this effect as being a negative one. Nevertheless, this number significantly is divergent between IBD subtypes, with only about one in five UC patients but almost every second CD patient stating, that coffee intake adversely affects course of their disease.

Interestingly, the negative patient’s perception of regular coffee intake on the course of disease did not lead to complete abstinence. Quite the contrary, a notably high fraction of patients (almost every second patient) perceiving coffee to negatively affect their course of disease did not refrain from regular coffee intake.

Our study has several limitations. With our unvalidated questionnaire we did not obtain specific data regarding disease localization, disease activity, medical therapy or data on quantitative average coffee intake as well as consume of other substances with or without caffeine such as tea and certain caffeine-containing soft-drinks or alcohol and cigarette smoking, respectively. However, the focus of this questionnaire was to give an impression about the frequency of coffee drinkers among a large number of unselected IBD patients and their perception of coffee on bowel disease. In order to achieve a number of replies as high as possible we decided to maximally constrict the amount of questions on the questionnaire, hence time needed for answering. Moreover, we decided to recruit patients only from the Swiss IBD patient organization representing the complete spectrum of IBD patients throughout Switzerland, thus minimizing a potential selection bias, that would likely occur if patients were recruited by their treating physicians or IBD nurses in larger hospitals or tertiary referral centers. Unfortunately, we believe, that such a selection bias may be an important confounder in several published survey studies, where patients from larger centers are notoriously overrepresented.

In view of the finding that our patients rather distinctively ascribe a negative effect to regular coffee consumption (if an effect is ascribed at all), drawing any conclusions derived from previous *in vitro* and *in vivo* research of coffee or specific coffee ingredients with regards to the potential direction and mechanisms underlying the interrelation of coffee and IBD with the current state of knowledge is challenging. On the one hand there are some studies that showed a pro-inflammatory effect of coffee in the intact human gut, with an activation of NF-ĸB [44, 45]. Moreover, the stimulatory effects on gastrointestinal motility [39] might simply increase stool frequency and thus adversely affect IBD symptoms. On the other hand, other previous findings rather suggest a protective efficacy of coffee in IBD through anti-inflammatory [41, 46, 47] and anti-neoplastic [29, 46, 48] properties.

Although the discrepancy between CD and UC patients is striking, this survey does not provide any guidance on the potential reasons behind the perceived stronger negative impact of coffee consumption in CD compared to UC. Nevertheless, it might be speculated, that differences in the localization, role of microbial composition, redox state, contribution of adaptive and innate immunity as well as inflammatory pathways most likely are underlying causative factors.

Evidently, a questionnaire-based survey cannot replace a mechanistic prospective investigation. Nevertheless, the results from various previous studies identifying effects of coffee ingredients on inflammation (some however with conflicting results) redox state, intestinal microbial composition and gastrointestinal motility in conjunction with the results from our survey corporately indicate an overall disease-modifying potential of coffee in general or one of its specific ingredients in IBD.

On the basis of our survey the question, why almost every second IBD patient who attributed a negative influence of coffee intake on the bowel symptoms, nevertheless regularly consumed coffee, cannot be answered. It may be speculated that the magnitude of the perceived effect on the disease course is only limited, not justifying or necessitating a complete abstinence. However, it might also be speculated, that the potential negative impact is indeed well perceived, but ignored or at least apportioned far less significance to. The latter would at least partly fulfill characteristics of an addiction, and in that be similar to active smoking in CD. Finally this high fraction of regular coffee consumers despite the perceived negative impact might also represent the current uncertainty on the exact role of coffee consumption on their symptoms due to the paucity of literature with regards to IBD, while the literature on coffee and a multitude of other gastrointestinal and general medical disease states has intriguingly increased and mostly suggested beneficial properties of coffee or specific coffee components [21–31].

Thus, these findings call for further controlled *in vitro* and *in vivo* studies to investigate the effect of coffee consumption in IBD as well as the observed divergence between CD and UC, including controlled trial in humans using clinical and biochemical endpoints.

## Conclusions

In conclusion, we showed that a substantial fraction of IBD patients identify regular coffee consumption as an impacting factor on their intestinal symptoms. An overall influence is significantly more often attributed to coffee in patients with CD. In addition, in these CD patients a significantly higher fraction is convinced that coffee adversely affects their intestinal symptoms compared with their counterparts with UC, where most patients do not identify a correlation between coffee and their disease. However, a negative perception on the effect of coffee on the course of disease, if present, does not appear to translate into an abstinence from coffee consumption.

## Additional file

Additional file 1: Table S1. Questionnaire. (DOCX 20 kb)

## Abbreviations

IBD: Inflammatory bowel disease
CD: Crohn’s disease
UC: Ulcerative colitis
IBDU: IBD unclassified
GIT: Gastrointestinal tract
SMCCV: Swiss Crohn’s and Colitis Union (*Schweizerische Morbus Crohn und Colitis Ulcerosa Vereinigung*)
