# Supplementary material for: Patients’ perceptions on the impact of coffee consumption in inflammatory bowel disease: friend or foe? – a patient survey
Source: Nutr J. 2015 Aug 12;14:78. doi: 10.1186/s12937-015-0070-8 (PMC4534065; doi:10.1186/s12937-015-0070-8)
Supplement: Additional file 1: Table S1. — Questionnaire. (DOCX 20 kb) [file 12937_2015_70_MOESM1_ESM.docx]

**SUPPLEMENTARY TABLE**

1. From which subtype of IBD do you suffer from?
   ☐ Crohn's disease
   ☐ Ulcerative colitis

☐ IBD unclassified /Colitis indeterminata

1. In case you drink coffee regularly - Do you drink it…

☐ Mostly caffeinated
☐ Mostly decaffeinated
☐ Don't know

1. In case you do not drink coffee regularly - Why not?
   ☐ Because of negative effects on my intestinal symptoms
   ☐ Because of another reason
   ☐ Don't know
2. Do you think, that coffee has an effect on the symptoms of your intestinal disease?

☐ Yes, positive effect
 ☐ Yes, negative effect
 ☐ No effect at all
 ☐ Don't know

Supplementary Table 1.: Questionnaire.
